# Supplementary material for: Complementary genetic and epigenetic changes facilitate rapid adaptation to multiple global change stressors
Source: Proc Natl Acad Sci U S A. 2025 Jul 15;122(29):e2422782122. doi: 10.1073/pnas.2422782122 (PMC12305003; doi:10.1073/pnas.2422782122)
Supplement: Supplementary file 1 — Appendix 01 (PDF) [file pnas.2422782122.sapp.pdf]

Supplementary Materials for: Complementary genetic and epigenetic changes facilitate rapid adaptation to multiple global change stressors

*Corresponding author. Email: reid.brennan@gmail.com; mpespeni@uvm.edu

Table S1: Results from gene ontology enrichment tests for each set of significant loci for OWA, warming, and acidification. Significant groups were determined with the weight method from TopGO. The ontology category is shown for each group. BP: Biological Process; CC: Cellular Component; MF: Molecular Function.

Treatment	GO_ID	Term	Annotated	Significant	Expected	classicFisher	weight	ontology
OWA	GO:0006310	DNA recombination	98	17	2.44	1.90E-11	0.00013	BP
OWA	GO:0032197	transposition, RNA-mediated	13	4	0.32	0.0002	0.0002	BP
OWA	GO:0021591	ventricular system development	6	2	0.15	0.0085	0.00852	BP
OWA	GO:0075732	viral penetration into host nucleus	6	2	0.15	0.0085	0.00852	BP
OWA	GO:0090630	activation of GTPase activity	7	2	0.17	0.0117	0.01174	BP
OWA	GO:0044826	viral genome integration into host DNA	8	2	0.2	0.0154	0.01541	BP
OWA	GO:0075713	establishment of integrated proviral lat...	8	2	0.2	0.0154	0.01541	BP
OWA	GO:0044264	cellular polysaccharide metabolic proces...	9	2	0.22	0.0195	0.02442	BP
OWA	GO:0046718	viral entry into host cell	12	2	0.3	0.0341	0.03411	BP
OWA	GO:0051056	regulation of small GTPase mediated sign...	36	3	0.9	0.058	0.03892	BP

OWA	GO:0030430	host cell cytoplasm	6	2	0.11	0.0049	0.0049	CC
OWA	GO:0042025	host cell nucleus	7	2	0.13	0.0067	0.0067	CC
OWA	GO:0044423	virion part	8	2	0.15	0.0089	0.0089	CC
OWA	GO:0000307	cyclin-dependent protein kinase holoenzy...	11	2	0.21	0.0169	0.0169	CC
OWA	GO:0008270	zinc ion binding	136	10	3.91	0.00413	0.0041	MF
OWA	GO:0008233	peptidase activity	83	14	2.39	2.40E-08	0.0074	MF
warming	GO:0006310	DNA recombination	98	7	0.76	2.30E-06	0.035	BP
warming	GO:0008233	peptidase activity	83	7	0.68	1.30E-06	0.0039	MF
warming	GO:0003887	DNA-directed DNA polymerase activity	13	2	0.11	0.00464	0.0046	MF
warming	GO:0004523	RNA-DNA hybrid ribonuclease activity	13	2	0.11	0.00464	0.0046	MF
acidification	GO:0006508	proteolysis	163	4	0.68	0.0023	0.0023	BP
acidification	GO:0006313	transposition, DNA-mediated	35	2	0.15	0.0082	0.0082	BP

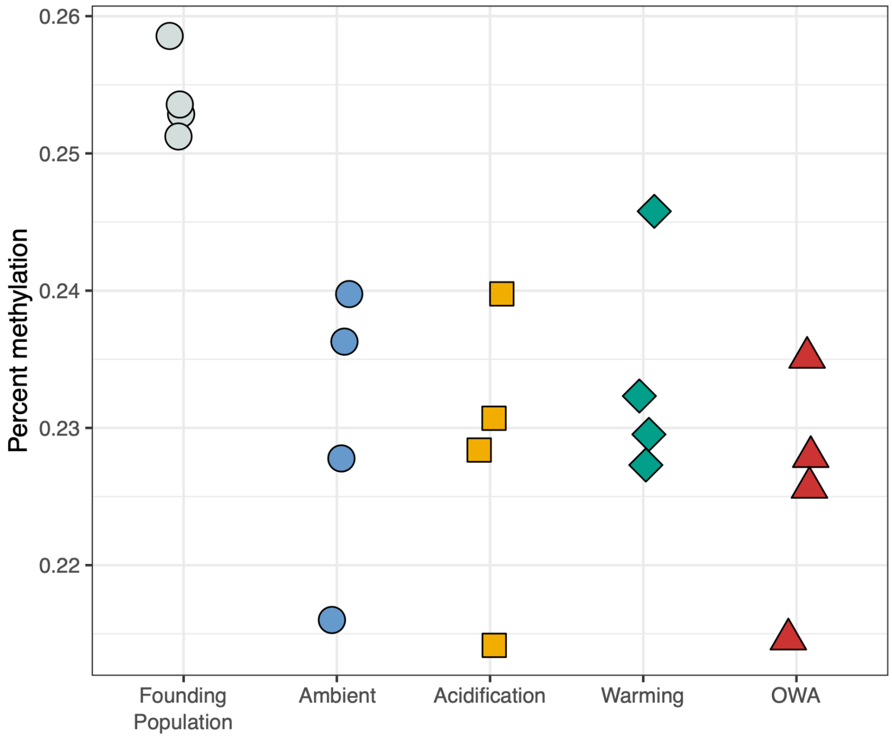

Fig. S1. Mean methylation percentage of all treatments. Each point is an individual replicate. The color and shape indicate treatment.

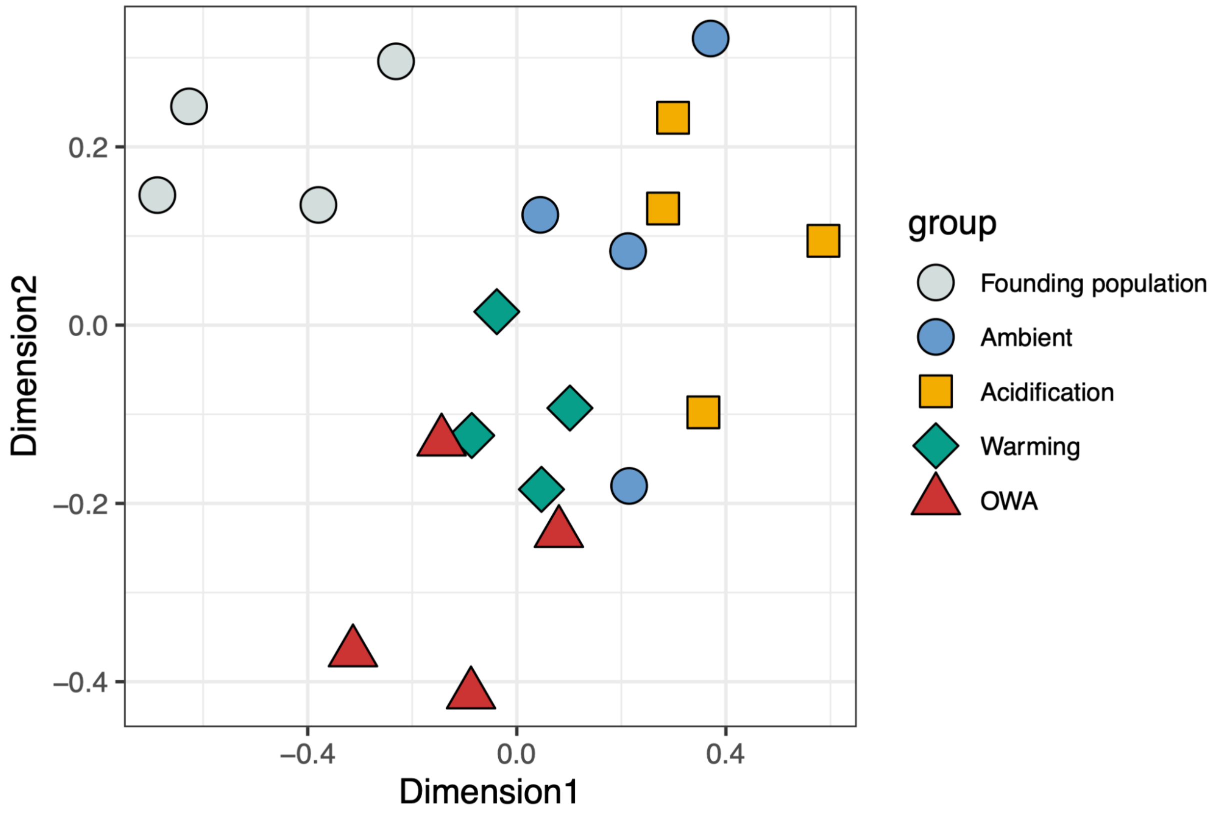

Fig. S2. Multidimensional scaling of methylation frequencies of 96,207 loci across replicate founding and selection populations.

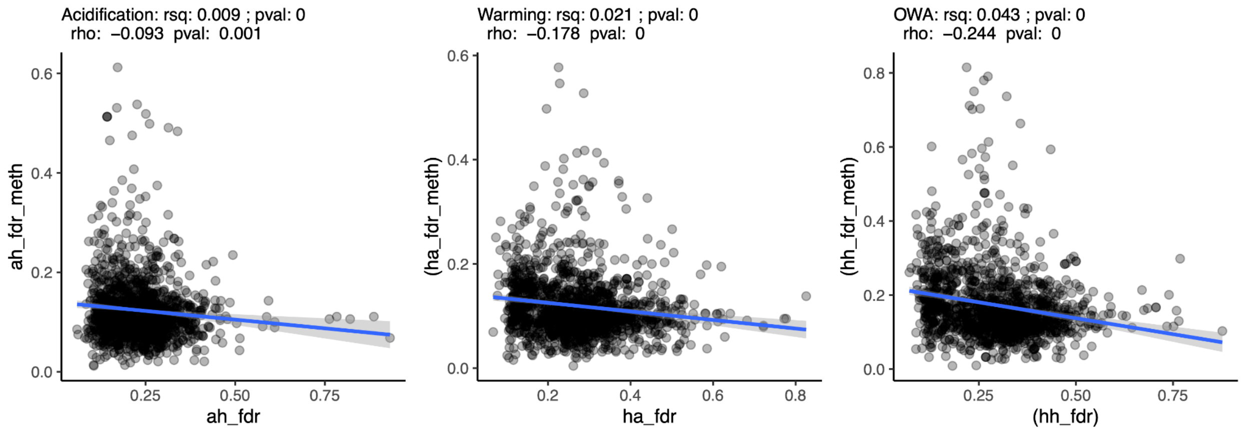

Fig. S3: Comparison of methylation and allele frequency divergence (1349 genes with at least 4 SNPs and 4 methylation sites). The blue line and statistics are from a linear regression. This relationship holds if the number of SNPs/methylation sites required is changed. Because assumptions of the regression are violated, non-parametric Spearman's rank correlation was also calculated: acidification: rho = -0.09, p = 0.0006; warming: rho = -0.18, p= 4.339e-11; OWA: rho = -0.24, p < 2.2e-16.

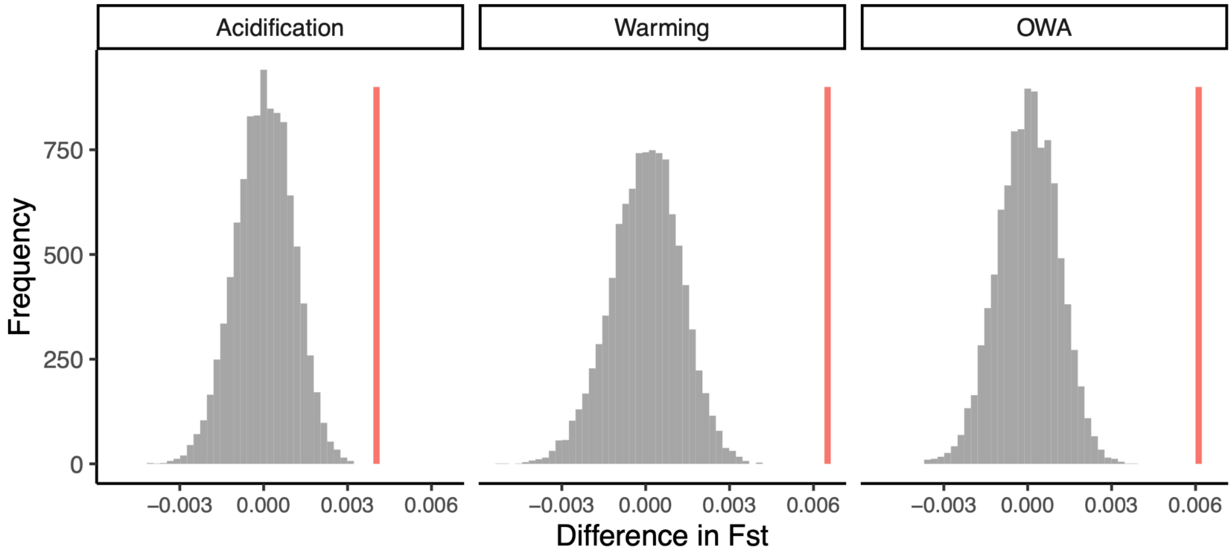

Fig. S4: Permutation test for the significance of the relationship between FST and methylation change. The genome was broken into 1.5 kb windows that contained at least 5 SNPs and 5 methylation sites, resulting in 910 windows across the genome. The vertical pink line is the observed difference in mean F_{ST} between windows containing significant versus non-significant methylation sites. To test if the observed difference in F_{ST} was greater than expected by chance, a permutation test (n=10,000) was run, randomizing the windows categorized as significant for methylation change and calculating the difference in F_{ST}. Resulting p-values from these permutations: $p_{acidification} = 0.0001$; $p_{warming} = 0.0001$; $p_{OWA} = 0.0001$. This permutation corresponds to Figure 3 in the main text.

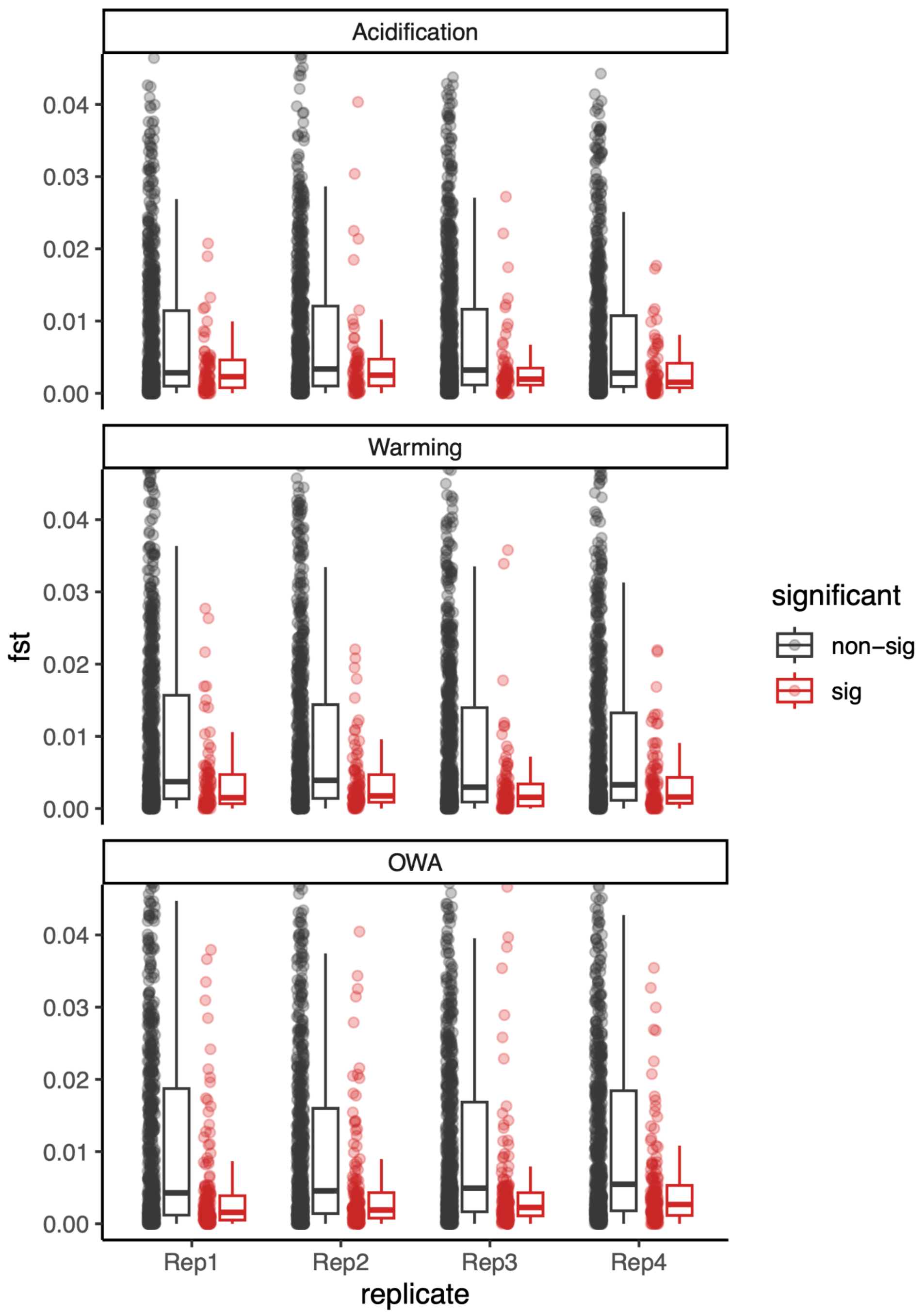

Fig. S5: Distribution of F_{ST} values in 910 1.5 kb windows for each replicate and treatment. Windows contained at least 5 methylation and 5 SNP loci. Red windows ("sig") are those with at least one significant methylation change. Black windows ("non-sig") contain no significant change.

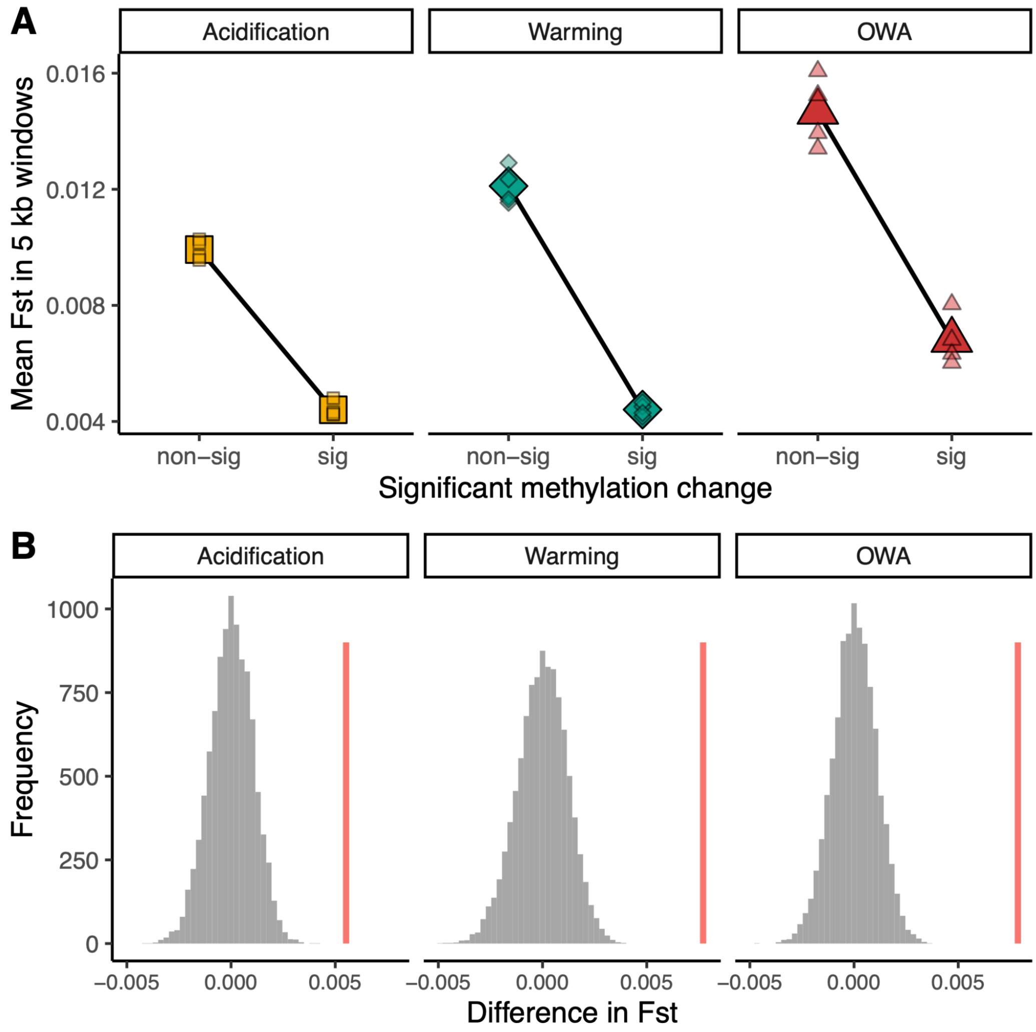

Fig. S6: Relationship between F_{ST} and methylation change for acidification, warming, and OWA treatments for 5kb window analysis; this analysis is the same as in Figure 2 but with a different window size. (A) The genome was broken into 5 kb windows that contained at least 5 SNPs and 5 methylation sites, resulting in 1102 windows. Each small point represents a replicate and the large symbols are the mean of all replicates. If a window contained at least one significant change in methylation from the ambient treatment, it was considered significant. (B) Permutation test (10,000) for the difference in F_{ST} observed for each treatment. The vertical pink line is the observed difference in mean F_{ST} between 5 kb windows containing significant versus non-significant methylation sites. Resulting p-values from these permutations: $p_{acidification} = 0.0001$; $p_{warming} = 0.0001$; $p_{OWA} = 0.0001$.

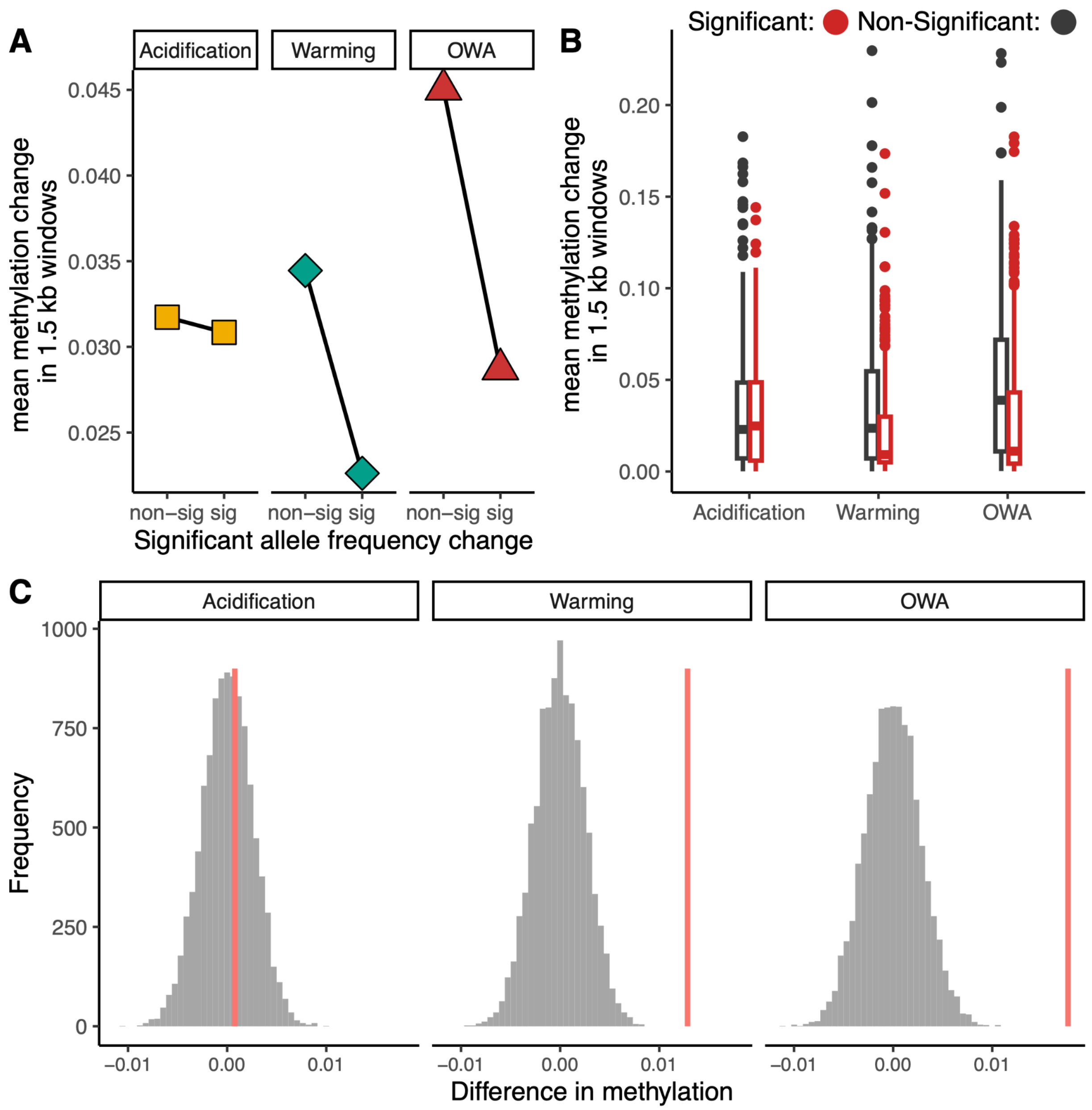

Fig. S7: Methylation changes for windows with significant allele frequency divergence. This is the opposite analysis from that presented in the main text. (A) The genome was broken into 1.5 kb windows that contained at least 5 SNPs and 5 methylation sites, resulting in 910 windows across the genome. Each point represents the mean methylation percentage. If a window contained at least one significant change in allele frequency from the ambient treatment, it was considered significant. (B) Distribution of mean methylation values shown in panel A. (C) Permutation test (10,000) for the difference in F_{ST} observed for each treatment. The vertical pink line is the observed difference in mean F_{ST} between 5 kb windows containing significant versus non-significant methylation sites. Resulting p-values from these permutations: $p_{acidification}$ = 0.3876; $p_{warming}$ = 0.0001; p_{OWA} = 0.0001.

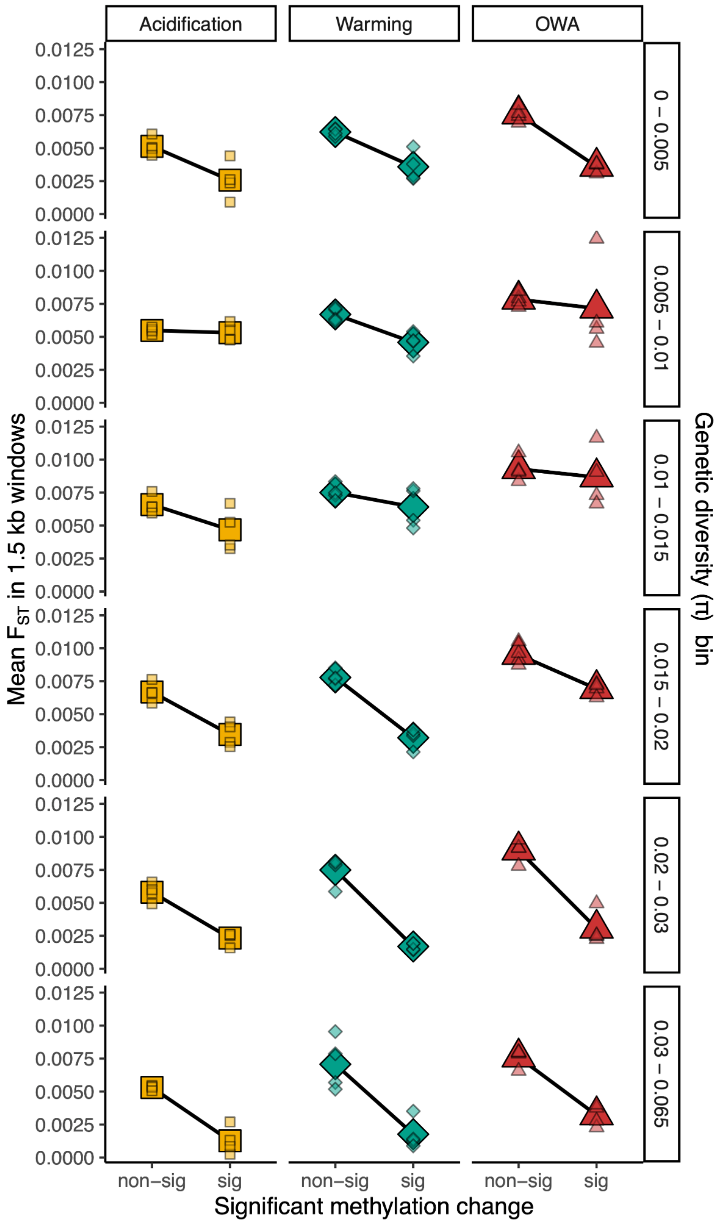

Fig. S8: Relationship between F_{ST}, methylation change, and genetic diversity. The genome was broken into 1.5 kb windows that contained at least 5 SNPs and 5 methylation sites, resulting in 910 windows across the genome. Genetic diversity (π) was estimated for 867 of these windows and each was assigned into bins based on π: 0-0.005 (n=103), 0.005-0.01 (n=165), 0.01-0.015 (n=173), 0.015-0.02 (n=173), 0.02-0.03 (n=170), > 0.03 (n=83). Each small point represents a replicate and the large symbols are the mean of all replicates. If a window contained at least one significant change in methylation from the ambient treatment, it was considered significant.

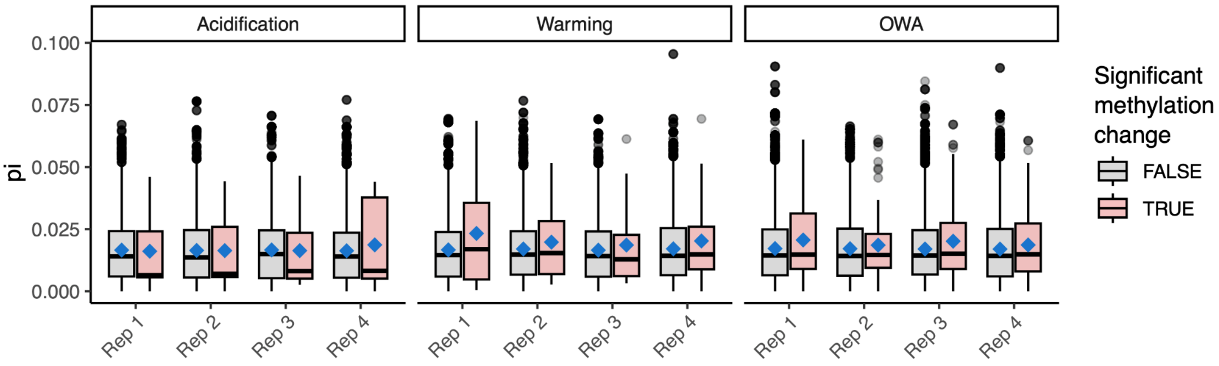

Fig. S9: Relationship between genetic variation and methylation changes. Tajima's π was calculated in 100 bp windows. All windows overlapping with a methylation locus were retained. Solid blue diamonds show the mean for each group and the horizontal lines show the median, as standard in a Tukey boxplot. For OWA, windows with significant methylation changes (TRUE) had significantly higher genetic diversity than those with no methylation change (FALSE; $p = 0.0001$; Kolmogorov-Smirnov Test). This pattern did not hold for acidification ($p = 0.2$) and warming ($p = 0.7$), though the number of windows overlapping with significant methylation changes was much lower than for OWA (acidification = 30; warming = 90; OWA = 456), which may lead to a poor ability to detect a signal.

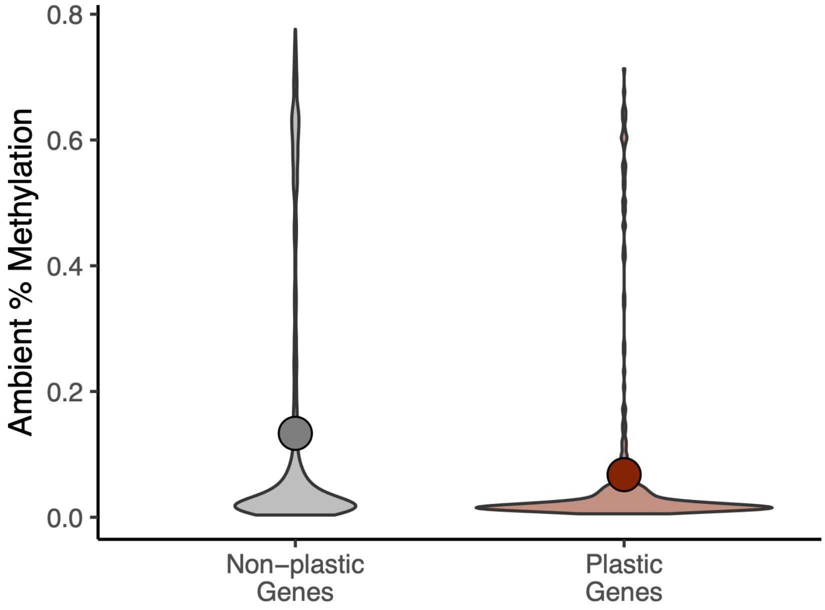

Fig. S10: Ambient plasticity genes vs. methylation. The percent methylation of plastic vs. non-plastic genes following transplant of the ambient line from ambient to OWA at generation F21. Solid points show the mean methylation percent for each group (mean methylation ± se: Non-plastic: 0.133 ± 0.006; Plastic: 0.068 ± 0.007; mean ± se). Non-plastic genes had significantly higher methylation percent than plastic genes ($p = 8.6 \times 10^{-8}$, Kolmogorov-Smirnov Test).

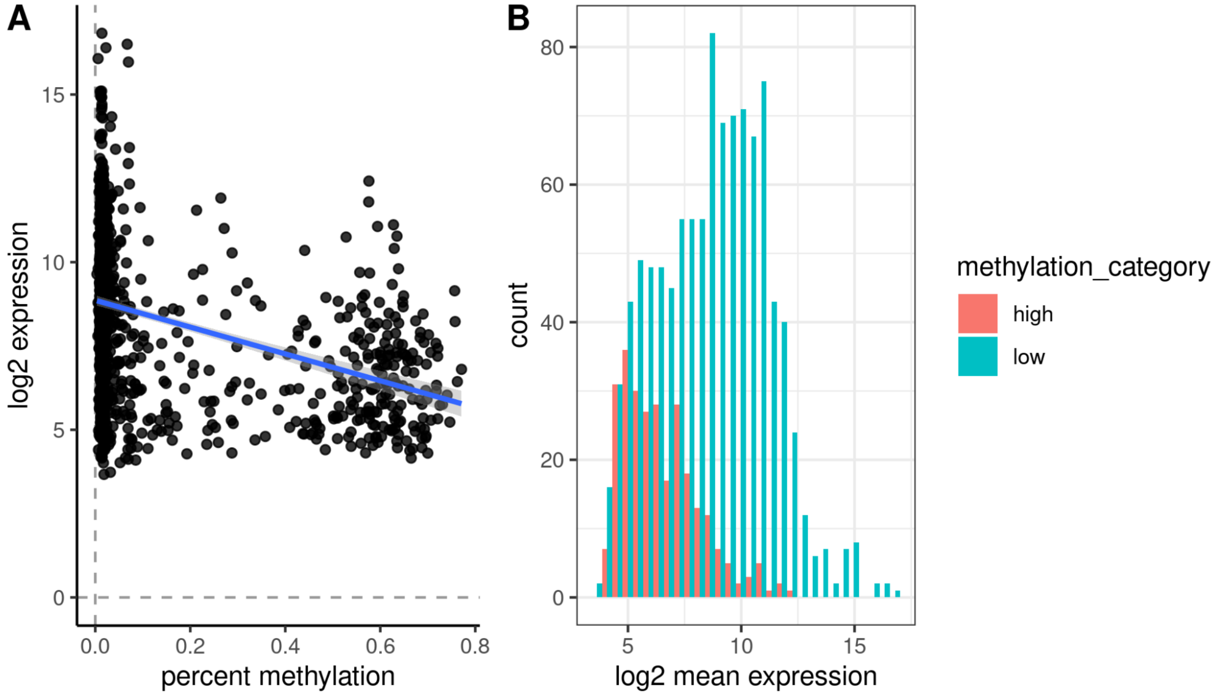

Fig. S11: Bimodal and negative relationship between methylation level and magnitude of gene expression. (A) A scatter plot where each point represents the methylation and expression level for a gene. (B) Histogram across log2 expression levels for genes categorized as having low (< 0.2%) or high (≥0.2%) methylation.

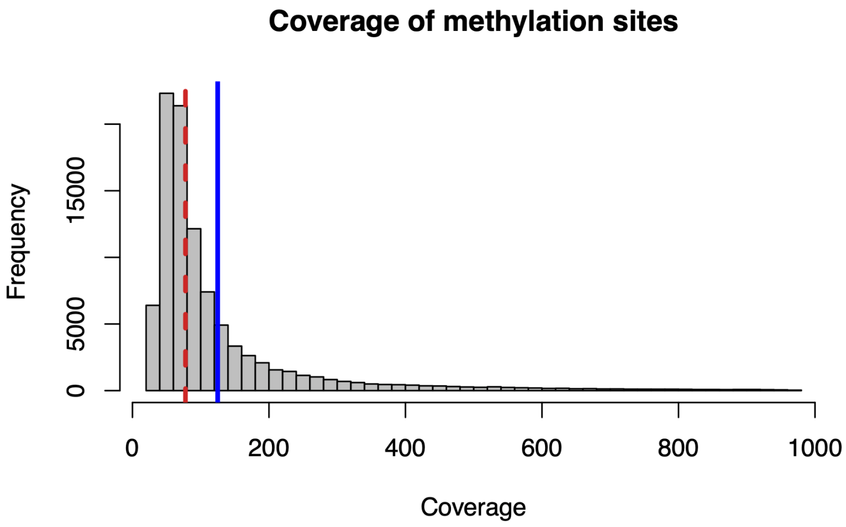

Fig. S12: Coverage histogram of methylated sites following filtering. The solid blue line is the mean coverage (124x) and the dashed red line is the median (77x).

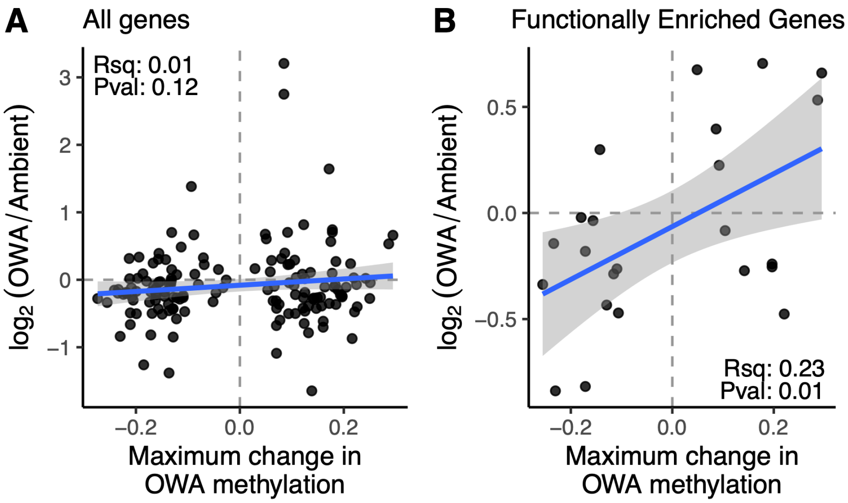

Fig. S13: Methylation and gene expression changes for methylation loci in exons only. (A) Divergence in gene expression between OWA and ambient lines versus the maximum change in OWA methylation from the ambient line for all overlapping genes (n=149). Genes were required to have at least 5 methylation sites with at least one significantly diverged methylation site in OWA. The solid blue line is the regression between these factors (R-squared = 0.01, p = 0.12; Spearman's rho = 0.09, p = 0.27). (B) Divergence in gene expression between OWA and ambient lines versus the maximum change in OWA methylation from the ambient line for genes with functional enrichment for methylation change (n=23). (R-squared = 0.23, p = 0.01; Spearman's rho = 0.35, p = 0.05).
